# Cerebrospinal fluid metabolite alterations associated with human brain aging: an untargeted metabolomics study

**DOI:** 10.3389/fmed.2026.1960863

**Published:** 2026-09-28

**Authors:** Yu Pan, He Ma, Qingqing Song, Bingjie Lai

**Affiliations:** 1 Department of Urology, The Second Hospital of Jilin University, Changchun, China; 2 Department of Anesthesiology, The Second Hospital of Jilin University, Changchun, China; 3 Department of Anesthesia, Critical Care and Pain Management, Massachusetts General Hospital, Harvard Medical School, Boston, MA, United States; 4 Department of Obstetrics and Gynecology, The Second Hospital of Jilin University, Changchun, China; 5 Department of Intensive Care Unit, The Second Hospital of Jilin University, Changchun, China

**Keywords:** aging, brain, cerebrospinal fluid, metabolites, untargeted metabolomics

## Abstract

**Background:**

Metabolic modifications underlying human brain aging still remains poorly investigated. This study aims to reveal the differential metabolite profile of cerebrospinal fluid (CSF) in the aging group, and demonstrate the metabolic drift in the cerebroventricular environment of human aging brain.

**Methods:**

Ten patients aged from 25 to 35 years old as the control group, and an identical number of patients aged from 70 to 80 years old as the aging group, who go through surgery without diagnosed aging-related neurological diseases, are enrolled in this study. CSF is collected prior to spinal anesthesia. Then, we conduct an untargeted metabolomics analysis of CSF samples, 10 specimens for each group, through using the Agilent 1,290 Infinity LC system. The stability of the instrument, the repeatability of the experiment, and the reliability of data are evaluated. Raw data are normalized and subjected to the subsequent analysis by R package (ropls). Differentially accumulated metabolites with a variable importance in projection greater than 1.0 are validated by Student's *t*-test.

**Results:**

A total of 1,041 metabolic features are obtained, and 33 differentially accumulated metabolites are identified from the control and aging groups. The instrument analysis system is tested to show a good stability, and acquired data are verified to be stable and reliable. The top three enriched chemical classifications of metabolites are lipids and lipid-like molecules, organic acids and derivatives, and organoheterocyclic compounds. Next, differentially accumulated metabolites are analysed under both patterns. Nepodin, Fa 18:2 + 1o, and L-threonate are significantly downregulated, and chlorophene, resorcinol, and isethionate are upregulated in the aging group compared to controls under the negative ion pattern. Notably, creatine, sarcosine, and N-stearoylsphinganine are upregulated in the positive ion pattern. Regulation of differentially accumulated metabolites is revealed. These differential metabolites are potentially involved in some overlapping pathways, including ABC transporters, protein digestion and absorption, and glycine, serine, and threonine metabolism.

**Conclusions:**

This untargeted metabolomics study identified age-associated differences in CSF metabolites, with enrichment in potential pathways such as ABC transporters and glycine, serine, and threonine metabolism. These preliminary findings require confirmation in larger, independently validated cohorts.

## Introduction

Human aging is a multidimensional process that reflects biological, psychological, and sociocultural impacts, and associated with many aging-related diseases such as neurodegenerative, cardiovascular, and metabolic disorders (1–3). As a complex course, aging affects various types of neuronal cells including neurons, astroglia, microglia and oligodendroglia, as well as connections in the brain. On the other hand, the functional maintenance of normal brain closely depends upon controlled energy metabolism, which is notable in the aged individuals or in those patients with aging-related neurodegenerative diseases (4). In terms of mechanisms, alterations of nucleotides, nucleic acids, proteins, lipids, carbohydrates, and metabolites, as parts of molecular substrates, result in impaired brain function in the aged brain (5). Of note, some differential metabolites may underlie micro-structural and functional brain changes in normal aging, which may give us a better understanding of brain aging (6).

Cerebrospinal fluid (CSF), as a culture medium that is present in the ventricles and spinal cord cavities as well as a complex solution that circulates around the central nervous system (CNS), provides a relatively stable cerebroventricular environment for the human brain (7, 8). Shuken and Wyss-Coray have revealed multiple changes of several proteins in abundance or structure during aging, as demonstrated by mass spectrometry analysis of CSF (9). In addition, Solvang and colleagues have reported kynurenine pathway metabolites derived from CSF are associated with human aging, and meanwhile identified a relatively stronger age-related upregulation of the potentially neurotoxic end-product in brain, i.e., quinolinic acid, offering robust evidence of changes in kynurenine metabolism with human aging and supporting links with adverse health outcomes (10). Recently, Liu et al. have identified thirty-seven metabolites in CSF with significant positive correlations with aging based on a Taiwanese population, such as cysteine, pantothenic acid, 5-hydroxyindoleacetic acid, aspartic acid, and glutamate (11). Therefore, metabolites in CSF are worth investigating in the context of brain aging.

CSF-based metabolomics, as a powerful tool in precision medicine, is a systematic study that allows us to track abundant different metabolites in CSF, and thereby better reflect the true pathophysiological state of individuals (12, 13). This technique has been applied to characterize brain tumors, transient focal cerebral ischemia-reperfusion injury, and Alzheimer's disease, etc (14–16). However, how differentially accumulated metabolites participate in the aging process of the brain remains poorly understood so far, which is still limited to some pilot studies (17, 18). Based on the background information, the main aim of this study is to reveal the differential metabolite profile of CSF in the aging group as well as the metabolic drift in the cerebroventricular environment of human aging brain through an untargeted metabolomics analysis, together providing preliminary clinical evidence that some important CSF metabolites are associated with brain aging.

## Materials and methods

All methods are carried out in accordance with relevant guidelines and regulations. Meanwhile, all experimental protocols are approved by the Second Hospital of Jilin University Institutional Review Board.

### Study population

From June 1, 2022 to December 31, 2022, a total of twenty adult participants, who are considered as cognitively healthy, i.e., without neurological or psychiatric diseases, and are undergoing optional spinal anesthesia for elective orthopedic surgery at the Second Hospital of Jilin University, China, are included in this study. G power analysis is used to estimate the sample size (effect size f²=0.35, *α*=0.05, power=0.80). Cognitive function is evaluated based on the preoperative assessment and the exclusion of neurological or psychiatric diseases. All participants are divided into the two groups by age: the control group (aged 25–35 years) and the aging group (aged 70–80 years). Differences in confounding factors including medication use, diet, lifestyle factors, and other potential confounders between the two groups are not significant (Supplementary Table S1). It is worth mentioning that the two-age group classification has been applied in the previous LC-MS metabolomics study of human aging (19). Given the fact that alterations in metabolites can be affected by insulin resistance, patients with diabetes (a fasting blood glucose > 126 mg/dL) and/or obesity (a body mass index > 30 kg/m^2^) are excluded from the final analysis. At last, 20 participants complete the initial screening and undergo CSF sampling for metabolomics analysis. Of note, all participants are admitted for elective orthopedic surgeries and fasted for ≥ 8 h before CSF sampling. We have explicitly explained the study protocol to every participant before enrolment into the study, and the written informed consent is obtained after explanation.

### CSF sample collection and preparation

The collection procedures of CSF samples are conducted in accordance with the previous metabolomics study published in 2021 (20). After obtaining the informed consent, we collect CSF samples during the routine spinal anesthesia procedure using a 26-gauge spinal needle at the L3–L4 or L4–L5 interspace. After free flow of clear CSF from the spinal needle, 1.2 mL of CSF is drained into a polypropylene tube, aliquoted, and stored at −80 °C until analysis. There is no immediate complications or patient discomfort reported during CSF sample collection. Then, 50 μL of CSF sample is mixed with 200 μL of cooled methanol to precipitate proteins. After centrifugation at 12,000 × g for 15 min, the supernatant is transferred to nitrogen gas for drying. The residue is suspended in 200 μL of 50% acetonitrile for liquid chromatography-mass spectrometry (LC-MS) analysis.

### Untargeted LC-MS analysis

Untargeted LC-MS analysis of CSF samples is performed by using an UHPLC (1,290 Infinity LC, Agilent Technologies) coupled to a quadrupole time-of-flight (AB Sciex TripleTOF 6,600). For hydrophilic interaction liquid chromatography (HILIC) separation, samples are analysed through using a 2.1 mm × 100 mm ACQUIY UPLC BEH Amide (1.7 μm) column (waters, Ireland). In both positive and negative electrospray ionization (ESI) patterns, the mobile phase contains A = 25 mM ammonium acetate and 25 mM ammonium hydroxide in water and B = acetonitrile. The gradient is 95% B for 0.5 min and is linearly reduced to 65% in 6.5 min, and then is reduced to 40% in 1 min and kept for 1 min, and then increased to 95% in 0.1 min, with a 3-min re-equilibration period employed.

The conditions of the ESI source are set as follows: Ion Source Gas1 (Gas1) as 60, Ion Source Gas2 (Gas2) as 60, curtain gas (CUR) as 30, source temperature as 600 °C, and IonSpray Voltage Floating (ISVF) as ± 5,500 V. In the acquisition phase of MS, the instrument is set to acquire over the m/z range 60–1,000 Da, and the accumulation time for TOF MS scan is set at 0.20 s/spectra. In the auto MS/MS acquisition phase, the instrument is set to acquire over the m/z range 25–1,000 Da, and the accumulation time for product ion scan is set at 0.05 s/spectra. The product ion scan is acquired using information dependent acquisition (IDA) with the high sensitivity mode. Specific parameters are set as follows: the collision energy (CE) is fixed at 35 V ± 15 eV, the decluttering potential (DP) is set at 60 V (+) and −60 V (−), the exclude isotopes within 4 Da, and the candidate ions to monitor per cycle:10.

### Data processing

Raw UHPLC-MS/MS data were processed using XCMS for peak alignment, peak picking, and quantification. Metabolite identification was performed by matching the acquired data against an in-house high-quality tandem mass spectral library (NovoMetDB), with a mass tolerance of 10 ppm and based on adduct ions. Background ions were removed according to blank sample signals, and relative peak areas were calculated as: Relative peak area = Sample raw value × (Sum of all sample raw values/ Sum of QC1 raw values). Metabolites with a coefficient of variation (CV) > 30% in QC samples were excluded. All data processing was conducted on a Linux system (CentOS 6.6) using R (3.4.3 and 4.0.3) and Python (2.7.6 and 3.5.0). Feature detection was performed with Compound Discoverer 3.3, and spectral matching was supplemented against the mzCloud, mzVault, and MassList databases. Metabolite annotation followed the Metabolomics Standards Initiative (MSI) confidence levels: level 2 for mzCloud/mzVault matches, and level 3 for MassList matches.

### Statistical analysis

After Sum and Pareto normalization, the processed data are analyzed by R package (ropls), where it is subjected to multivariate data analysis, including orthogonal partial least-squares discriminant analysis (OPLS-DA). In the positive ion pattern, R^2^X is 0.146 cum, R^2^Y is 0.94 cum, Q^2^ is 0.0629 cum; in the negative ion pattern, R^2^X is 0.237 cum, R^2^Y is 0.933 cum, Q^2^ is 0.235 cum. The 7-fold cross-validation and response permutation testing is used to evaluate the robustness of the model. The variable importance in the projection (VIP) value of each variable in the OPLS-DA model is calculated to indicate its contribution to the classification. Student's t-test is applied to determine the significance of differences between two groups of independent samples. Fold-change > 1.5, uncorrected *P* value < 0.05, VIP scores > 1.0 from PLS-DA are used to screen differentially accumulated metabolites. Pearson's correlation analysis is performed to determine the correlation between two variables.

## Results

### Chemical classifications and attribution statistics of identified metabolites

The instrument analysis system has been verified to display a good stability, and the acquired data are confirmed to be stable and reliable. Therefore, differences in the metabolic spectrum shown in this experiment can reflect the biological differences associated with the development of normal brain aging between these samples from the two groups.

It turns out that a total of 1,041 metabolites are identified, including 613 metabolites in the positive ion pattern and 428 metabolites in the negative ion pattern, respectively. All metabolites identified in this study are classified and statistically analysed based on their chemical taxonomy attribution information. Through analysing the positive and negative ion patterns respectively, the specific chemical classification and attribution information of each metabolite is detailedly shown. The proportions of the number of various metabolites are shown in Figure 1. The top five enriched chemical classifications of these metabolites are lipids and lipid-like molecules (22.863%), organic acids and derivatives (17.771%), organoheterocyclic compounds (16.907%), benzenoids (13.256%), and undefined metabolites (9.798%), suggesting the variety of metabolites that are differentially accumulated in CSF and probably involved in brain aging.

**Figure 1:**
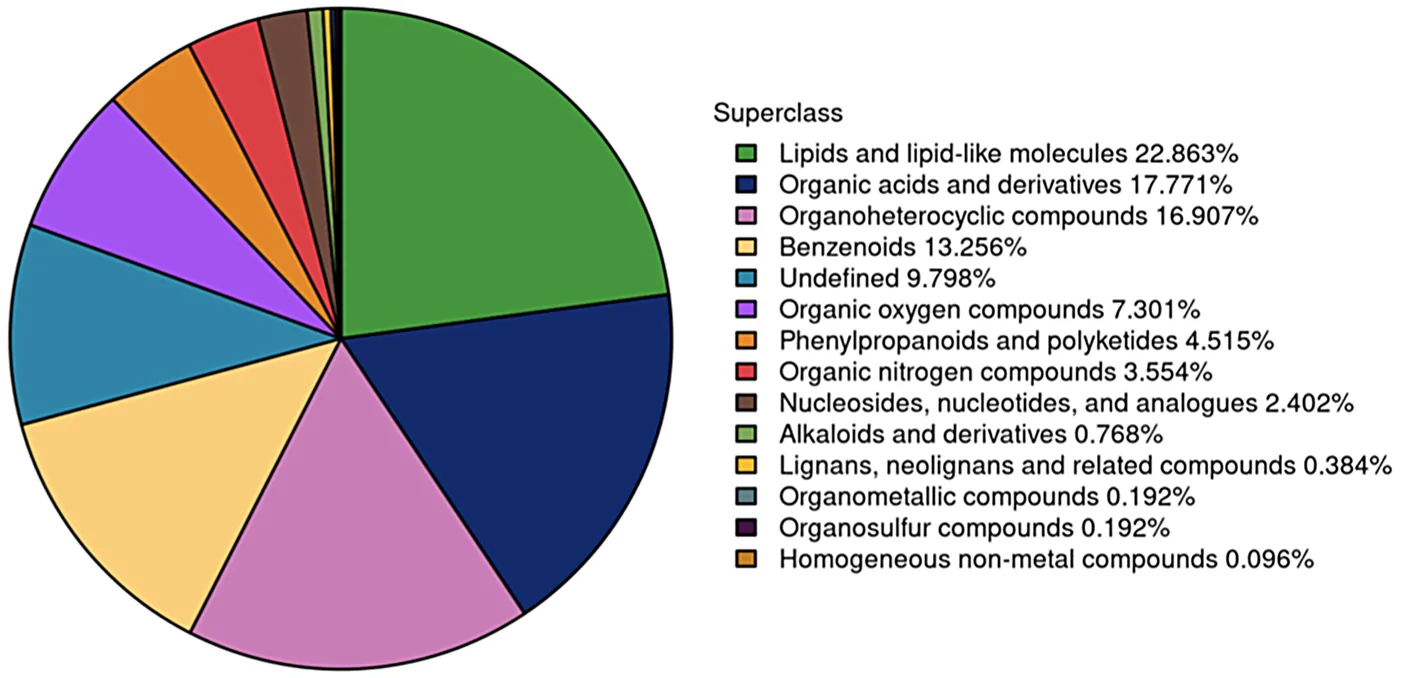
The proportion of identified metabolites derived from cerebrospinal fluid in various chemical classifications. The top four enriched chemical classifications of metabolites are lipids and lipid-like molecules (22.863%), organic acids and derivatives (17.771%), organoheterocyclic compounds (16.907%) and benzenoids (13.256%).

### Overview of differentially accumulated metabolites in the aging group

Then, differentially accumulated metabolites (FC > 1.5 or FC < 0.67, uncorrected *P* value < 0.05) in the aging group compared to the control group are displayed in volcano plots under both ion patterns (Figures 2A,B). Red dots represent upregulated metabolites, blue dots indicate downregulated metabolites, and black dots represent those metabolites without the differential expression.

**Figure 2:**
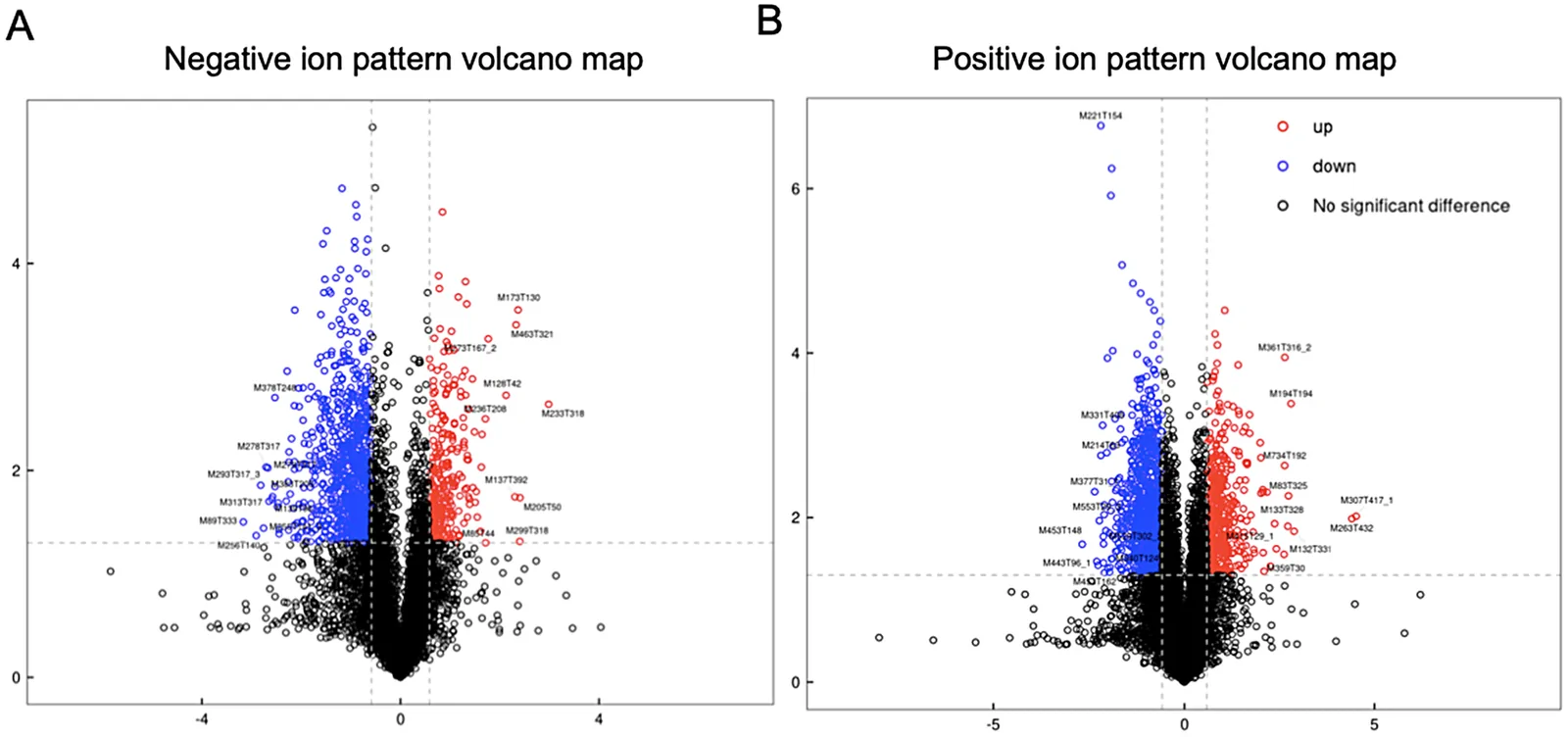
Overview of extracted metabolites. **(A)** Representative volcanic map showed detected metabolites under the negative ion pattern. **(B)** Representative volcanic map showed detected metabolites under the positive ion pattern. Red dots represent up-regulated metabolites. Blue dots indicate downregulated metabolites. Black dots represent metabolites without differential expression.

The top nine downregulated metabolites in the negative ion pattern are Nepodin, Fa 18:2 + 1o, L-threonate, dimethylglycine, glycolate, homocarnosine, D-psicose, 3-methyl-2-oxopentanoate, and L-aspartic acid (Figure 3A). In contrast, the top nine upregulated metabolites under the negative ion pattern include chlorophene, resorcinol, isethionate, phytanic acid, orcinol, citramalic acid, octadecanoic acid, palmitic acid, and rhamnetin (Figure 3B). The top nine downregulated metabolites under the positive ion pattern are didodecyl 3,3'-thiodipropionates, syringic acid, alpha-d-mannose pentaacetate, zerumbone, leucine, L-propionylcarnitine, allose, tebutam, and diethylcarbamazine (Figure 3C). The top seven upregulated metabolites under the positive ion pattern are creatine, sarcosine, N-stearoylsphinganine, N-acetyl-l-aspartic acid, alisol a, 3-phosphonoalanine, and 3,5-dibromo-l-tyrosine in old people compared to controls (Figure 3D).

**Figure 3:**
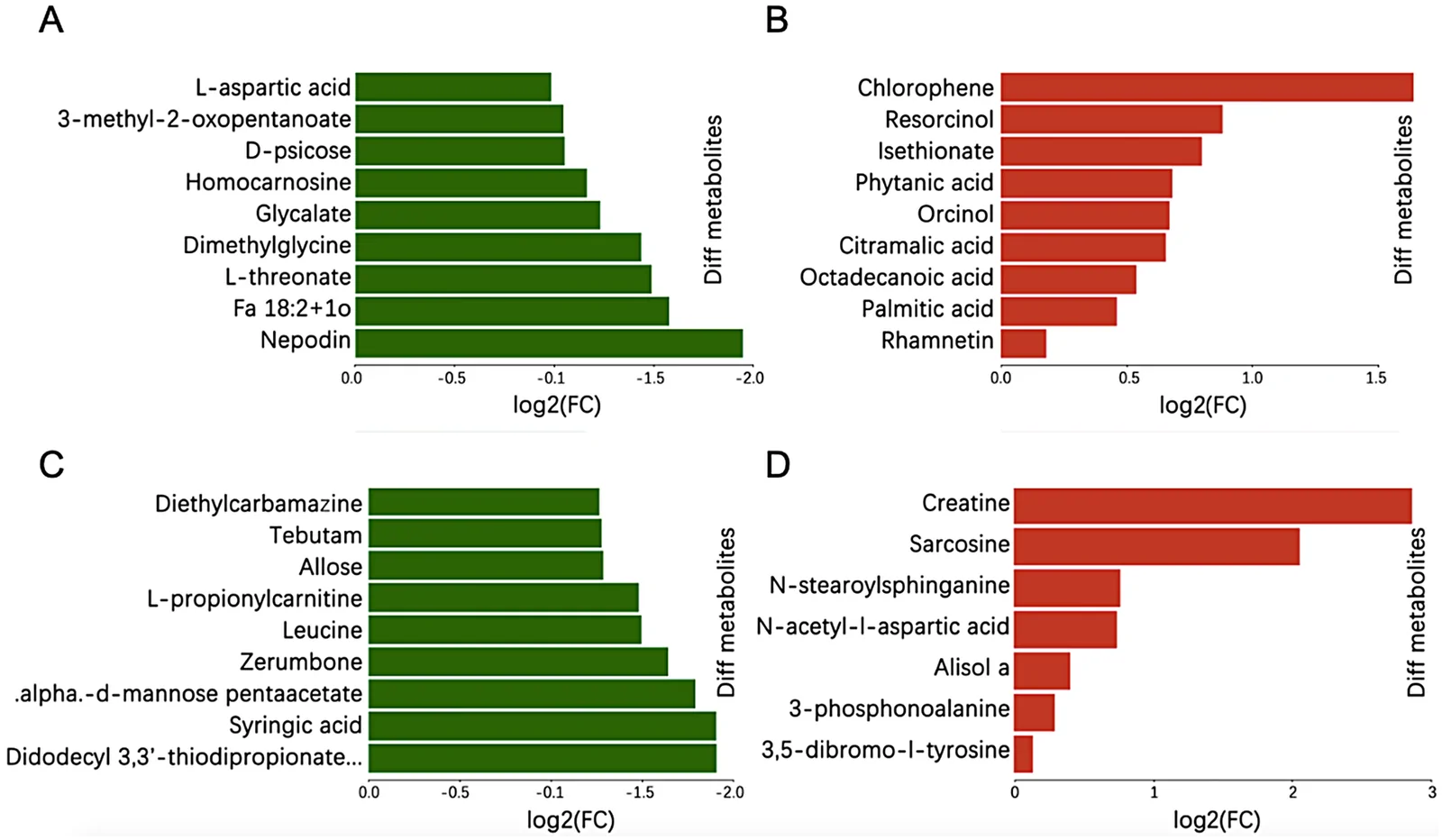
Differential metabolites under the negative ion pattern and the positive ion pattern. **(A)** The top nine downregulated metabolites under the negative ion pattern ion pattern, including nepodin, Fa 18:2 + 1o, L-threonate, dimethylglycine, glycolate, homocarnosine, D-psicose, 3-methyl-2-oxopentanoate, and L-aspartic acid. **(B)** The top nine upregulated metabolites under the negative ion pattern, including chlorophene, resorcinol, isethionate, phytanic acid, orcinol, citramalic acid, octadecanoic acid, palmitic acid, and rhamnetin. **(C)** The top nine downregulated metabolites under the positive ion pattern, including didodecyl 3,3’-thiodipropionates, syringic acid, alpha-d-mannose pentaacetate, zerumbone, leucine, L-propionylcarnitine, allose, tebutam, and diethylcarbamazine. **(D)** The top seven upregulated metabolites under the positive ion pattern, including creatine, sarcosine, N-stearoylsphinganine, N-acetyl-l-aspartic acid, alisol a, 3-phosphonoalanine, and 3,5-dibromo-l-tyrosine.

Furthermore, hierarchical heat maps show differentially accumulated metabolites in each patient under the negative and positive ion patterns, respectively (Figures 4A,B), indicating the individual differences in general.

**Figure 4:**
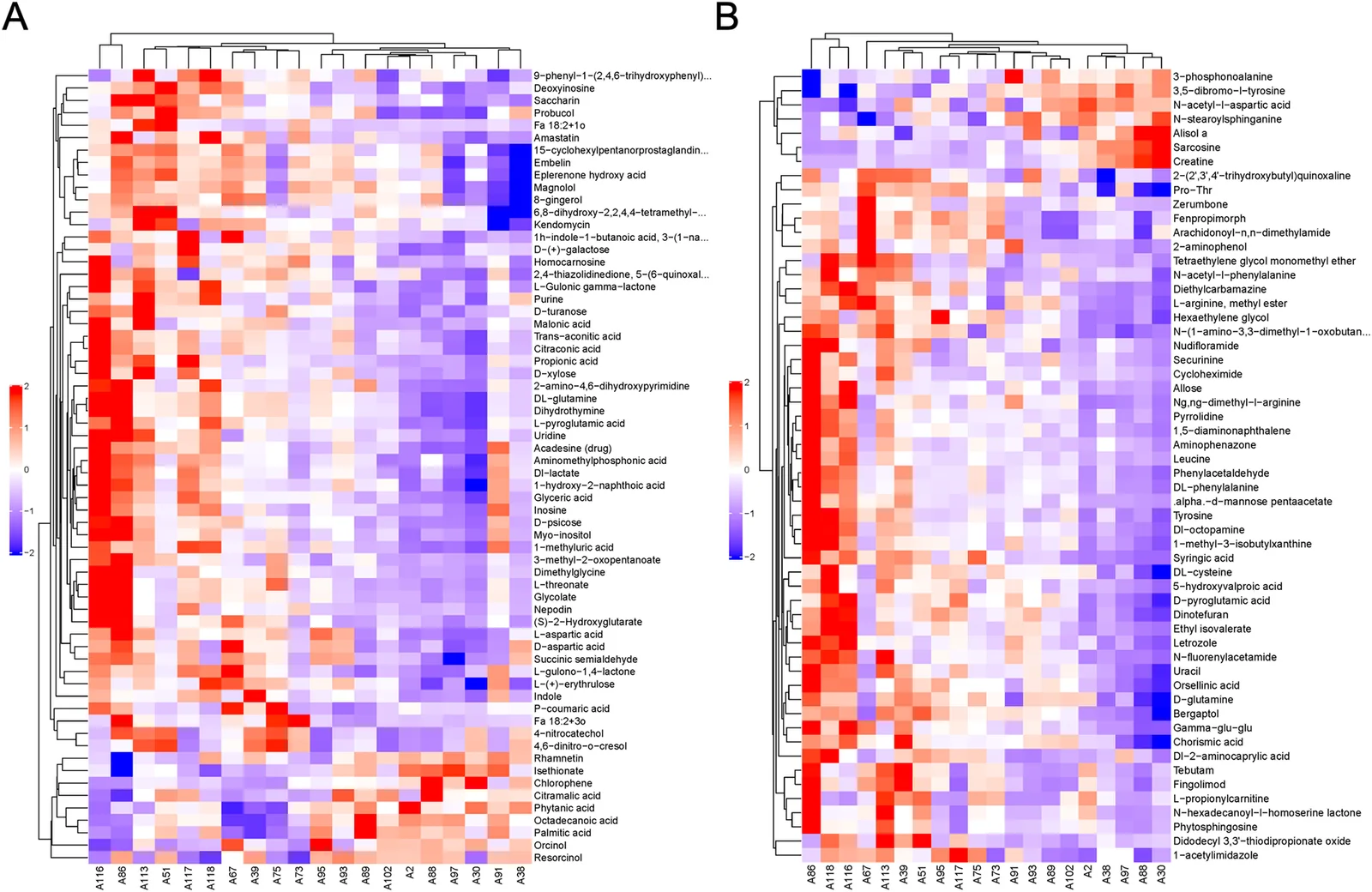
Hierarchical heat map of differentially accumulated metabolites. **(A)** Hierarchical heat map of differentially accumulated metabolites under the negative ion pattern (A70–80 Vs A25–35). **(B)** Hierarchical heat map of differentially accumulated metabolites under the positive ion pattern (A70–80 Vs A25–35).

### Regulation of differentially accumulated metabolites

Metabolites, comprising the largest fraction of molecules in cells, regulate each other in the development of brain aging (21). Therefore, it is essential to evaluate metabolite correlations to have a better understanding of their mutual regulation relationship further. Chord diagrams are used to display the correlation between metabolite superclass, including lipid and lipid molecules, nucleosides and analogues, organic acids and derivatives, organic oxygen compounds, organoheterocyclic compounds, and undefined metabolites, under the negative and positive ion pattern, respectively (Figures 5A, 6A).

**Figure 5:**
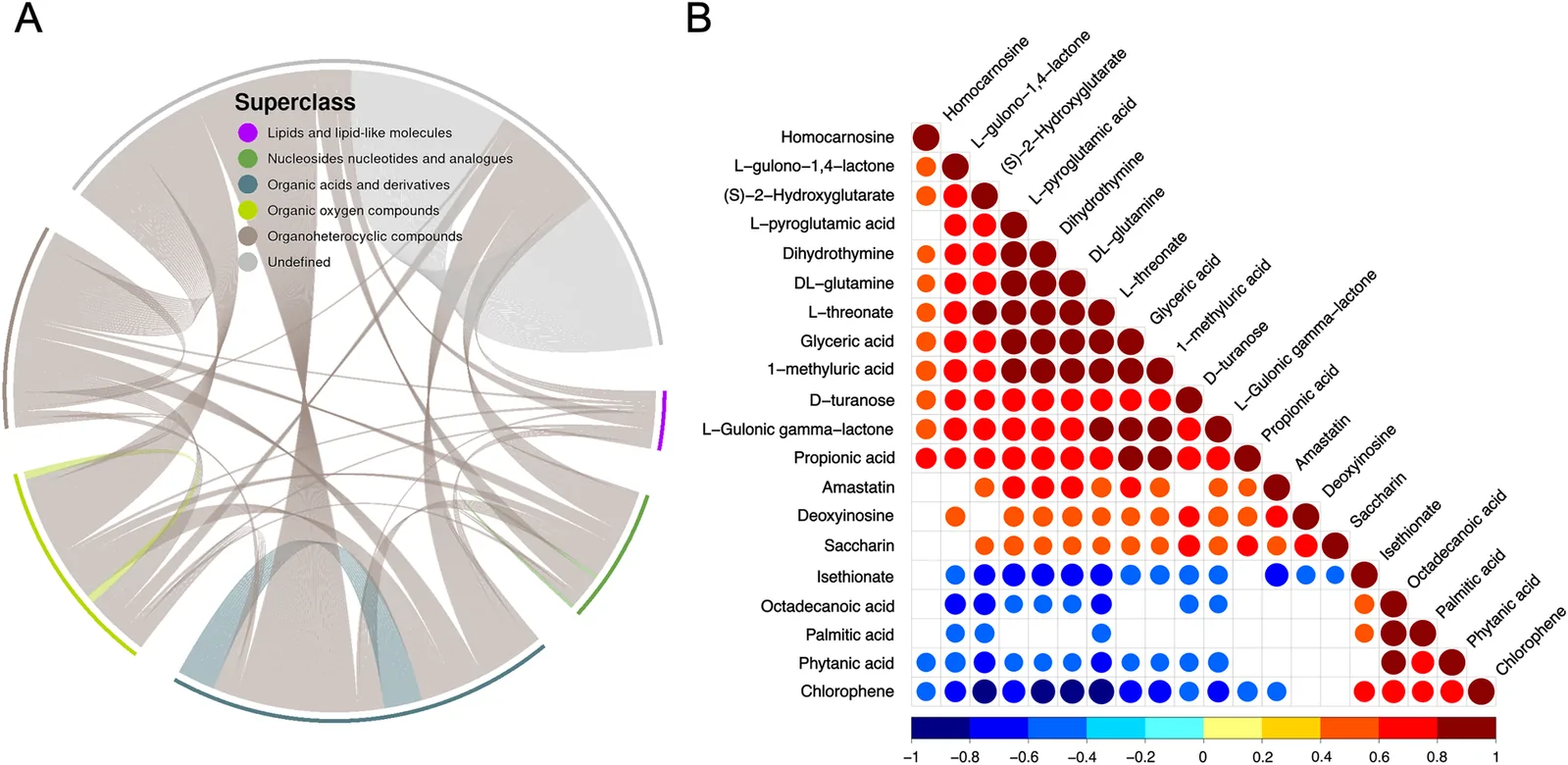
Regulation of differentially accumulated metabolites under the negative ion pattern. **(A)** Chord diagrams. Correlation heat map. **(B)** Metabolic proximities. The blank spaces (missing chords) represent non-significant correlations between metabolite classes.

**Figure 6:**
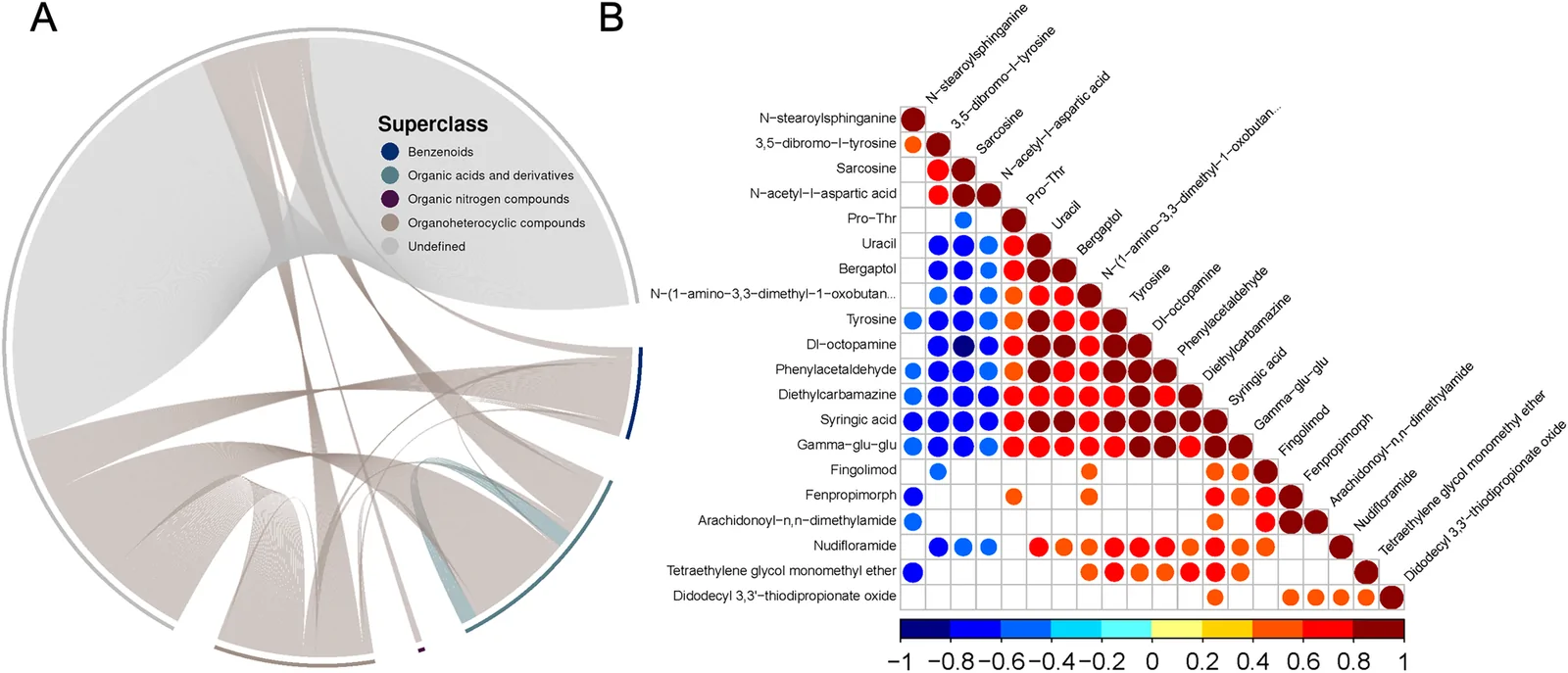
Regulation of differentially accumulated metabolites under the positive ion pattern. **(A)** Chord diagram. Correlation heat map. **(B)** Metabolic proximities. The blank spaces (missing chords) represent non-significant correlations between metabolite classes.

Meanwhile, correlation heat maps are used to assess metabolic proximities of specific differentially accumulated metabolites (VIP>1，*P* value < 0.05) under both the two patterns (Figures 5B, 6B). In the negative ion pattern, chlorophene is negatively correlated with homocarnosine, L-gulono-1,4-lactone, (S)-2-hydroxyglutarate, L-pyroglutamic acid, dihydrothymine, DL-glutamine, L-threonate, glyceric acid, 1-methyluric acid, D-turanose, L-gulonic gamma-lactone, propionic acid, and amastatin, whereas positively correlated with isethionate, octadecanoic acid, palmitic acid, and phytanic acid (Figure 5B). In the positive ion pattern, tyrosine is negatively correlated with N-stearoylsphinganine，3,5-dibromo-l-tyrosine, sarcosine, and N-acetyl-l-aspartic acid, whereas positively correlated with pro-thr, uracil, bergaptol, and N-(1-amino-3,3-dimethyl-1-oxobutan-2-yl)-1-butyl-1H-indazole-3-carboxamide (Figure 6B).

### KEGG pathway analysis of differentially accumulated metabolites

Some Kyoto Encyclopedia of Genes and Genomes (KEGG) pathways in which differentially accumulated metabolites are enriched have been shown in Figure 7A. As displayed in the heat map, these differential metabolites are potentially involved in some overlapping pathways, including ABC transporters, protein digestion and absorption, and glycine, serine, and threonine metabolism. At last, the hierarchical heat map shows differently enriched KEGG pathways in each patient of the two groups, uncovering the individual differences (Figure 7B).

**Figure 7:**
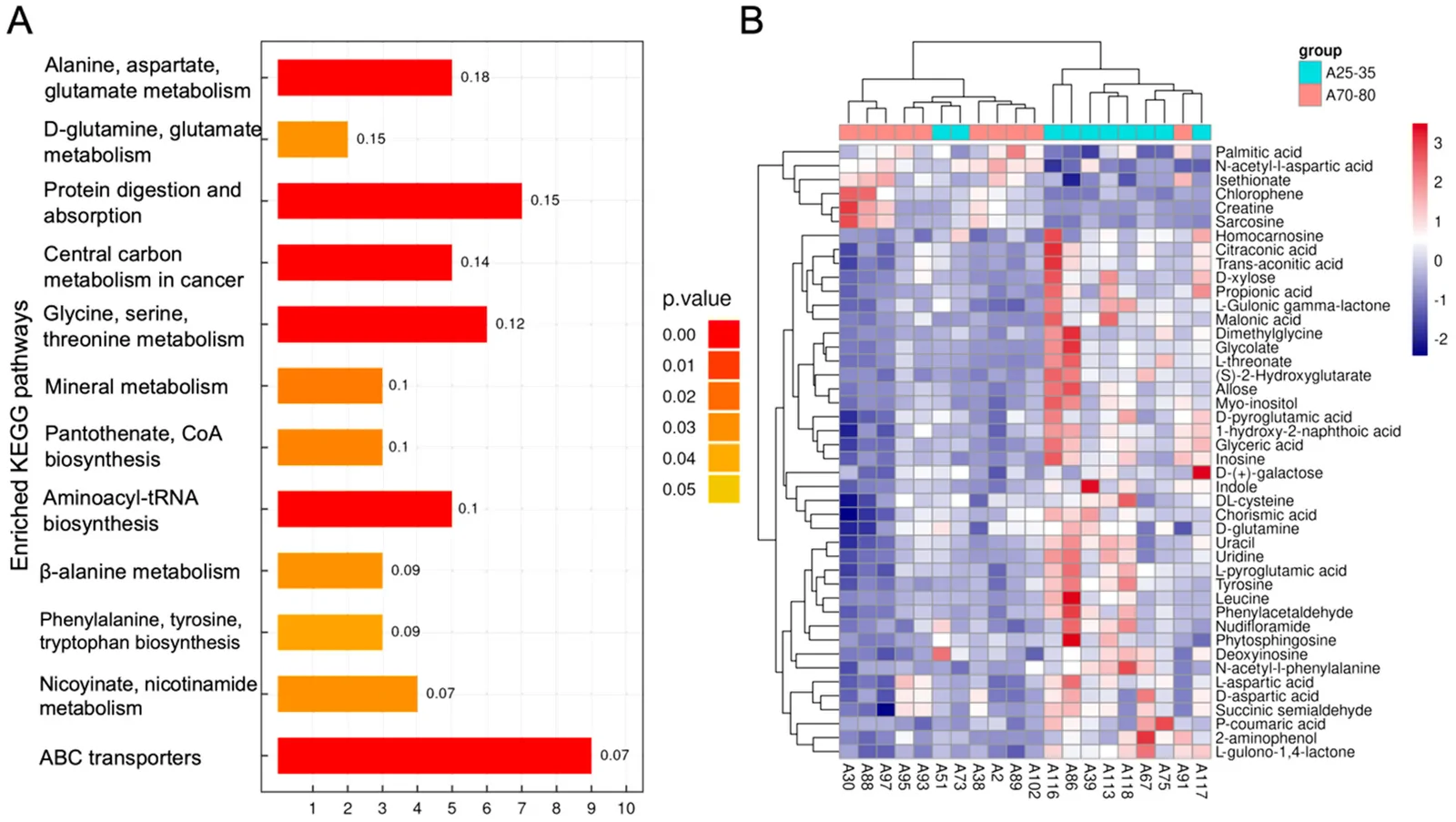
KEGG pathway analysis. **(A)** Heat map of KEGG pathway of differentially expressed metabolites. **(B)** KEGG enrichment pathway diagram of differentially accumulated metabolites.

## Discussion

It has been well acknowledged that metabolism dysfunction is tightly associated with brain aging, and thereby contributes to aging-related diseases (22, 23). Interestingly, brain aging is in turn affected by altered metabolites both in the blood circulation and cerebrospinal fluid (CSF) (24–26). CSF metabolic concentrations do not directly reflect the cerebroventricular environment, as they are subject to protein binding, transport kinetics, and blood–CSF barrier permeability. Our single-point sampling cannot capture dynamic flux. Therefore, the observed alterations should be interpreted as exploratory associations rather than quantitative measures of brain metabolism. As emphasized by Crudele et al., matrix-specific interpretation is critical when using CSF data. We recommend future validation in complementary matrices and dynamic sampling (27). However, there have been few relevant studies on the mechanistic understanding of normal brain aging in the field of metabolomics. Therefore, this study aims to systematically evaluate the metabolic proximities of those differentially accumulated metabolites in CSF obtained from the aged people and the controls through an untargeted metabolomics analysis. In our study, 1,041 metabolic features are detected, and 33 differentially accumulated metabolites are identified in old people (Supplementary Table S2). The top three enriched chemical classifications of metabolites are lipids and lipid-like molecules, organic acids and derivatives, and organoheterocyclic compounds. Moreover, these differential metabolites are potentially involved in some overlapping pathways, including ABC transporters, protein digestion and absorption, and glycine, serine, and threonine metabolism. Although other molecules are not discussed here, they may also exert an important role.

Among various kinds of metabolites, lipids and lipid-like molecules are one of the most important classes in nervous tissue with high structural and functional diversity, which certainly contribute to the structure, development, and function of healthy brains (28, 29). Furthermore, dysregulated lipid metabolism is linked to aging and diseased brains (30–32). Thus, characterization of lipid changes in the aging brain is helpful to improve our understanding of the molecular basis of brain aging, uncovering novel mechanisms underlying age-associated neurological diseases, and further offering favourable opportunities for therapeutic intervention (33, 34). Mora and colleagues have summarized that an increased level of docosahexaenoic acid (DHA) and eicosapentaenoic acid (EPA) in the elderly is closely associated with improved cognitive function and a lower risk of dementia, even though consistent clinical evidence is still lacking (35). More recently, a class of anti-inflammatory lipids, i.e., 3-sulfogalactosyl diacylglycerols (SGDGs), have been identified to be decreased with aging in the central nervous system (CNS), which are probably associated with myelination (34). In terms of the specific mechanism, an SGDG was found to inhibit LPS-induced gene expression and release of pro-inflammatory cytokines from macrophages and microglia through the NF-*κ*B pathway, highlighting the brain lipidome's complexity and potential biological functions in aging (34). In summary, lipids and lipid-like molecules are potent metabolites in brain aging, which need more attention in future research.

Moreover, changes in amino acid profiles play a key role in aging process, as revealed by recent CSF research. A metabolomics study from Barzegar Behrooz and colleagues have detected decreased levels of many kinds of amino acids, including alanine, arginine, aspartic acid, glutamic acid, glycine, isoleucine, methionine, phenylalanine, proline, serine, threonine, tryptophane, tyrosine, and valine, in CSF of Alzheimer's disease-like animals as compared to controls (36). Solvang and coworkers have demonstrated that aging activates the inflammation and stress-driven kynurenine pathway systemically and in the brain (10). Lin et al. have identified increased levels of alanine, citrate, creatinine, lactate, leucine, tyrosine, and valine in old patients compared to those in young patients (20). Similarly, Peters et al. have found perturbations in the tryptophan and kynurenine pathways in CSF of aged subjects (37). In this study, we have also observed upregulated creatine in CSF of aged people. It has been well studied that creatine is a key metabolite implicated in Alzheimer's disease and mild cognitive impairment (38, 39). In aging, increases in choline and creatine may suggest glial proliferation (40). Taken together, amino acid metabolism is often associated with aging.

In addition to lipids and lipid-like molecules and amino acids, age-dependent changes are observed in many other molecules, such as organic acids and derivatives, and organoheterocyclic compounds, as reported by Chen, Seo and co-workers (41, 42). This may hold true for the pathology of normal brain aging. Based on a Taiwanese population, data from Liu et al. have revealed several significantly altered CSF metabolites in amino acid metabolism during aging and between the sexes through LC-MS metabolomics (11). These metabolic alterations in CSF may provide clues for healthy brain aging and deserve further exploration. In a similar vein, our results from this study for the first time give the clinical evidence that organic acids and derivatives, and organoheterocyclic compounds are the most enriched chemical classifications of metabolites in CSF of the elderly, which supports to understand the association between the state of physiological aging and altered metabolism of organic acids and organoheterocyclic compounds.

It is worth mentioning that possible interactions of differentially accumulated metabolites have been elucidated in this study, given the fact that metabolites regulate each other, and contribute to the development of aging (43, 44). We have utilized chord diagrams to display the correlation between metabolite superclass, encompassing lipid and lipid molecules, nucleosides and analogues, organic acids and derivatives, organic oxygen compounds, organoheterocyclic compounds, and undefined metabolites, under the negative and positive ion pattern, respectively. Several differentially accumulated metabolites were functionally connected within enriched pathways. Sarcosine and creatine are both related to glycine metabolism: sarcosine derives from glycine, and glycine is a precursor for creatine synthesis; their concurrent upregulation suggests a coordinated shift in glycine-related and one-carbon metabolism. L-threonate, a vitamin C metabolite, was downregulated, possibly reflecting reduced antioxidant capacity. N-stearoylsphinganine belongs to the sphingolipid pathway, consistent with broader membrane lipid remodeling involving ABC transporters. Although these metabolites did not form a single linear cascade, they converge on amino acid metabolism, antioxidant defense, and lipid transport. Given the cross-sectional design, these relationships should be viewed as correlative rather than causal.

Afterwards, KEGG pathway analysis is performed to show possible function pathways in which differentially accumulated metabolites are probably involved (20, 45). These differential metabolites identified in the elderly have been predicted to be involved in some overlapping pathways, including ATP binding cassette (ABC) transporters, protein digestion and absorption, and glycine, serine, and threonine metabolism, etc. Of note, amino acid metabolism is a key metabolic pathway across brain aging (46, 47). Islam and Behura have revealed that *Cav1* deficiency leads to deregulation in the lipid and amino acid metabolism, and meanwhile genes associated with these deregulated metabolites have been found to be altered in the brain upon aging (46). As shown in this study, the precise metabolomics approach can reveal perturbations of differentially accumulated metabolites in a diverse array of metabolic pathways, which will be will be useful for understanding normal aging and aging-related diseases.

We further compared our findings with published metabolomic and metabolic studies of brain aging and age-related cognitive decline. Among the identified metabolites, N-stearoylsphinganine is consistent with broader sphingolipid alterations reported in Alzheimer's disease metabolomics (48), and L-threonate may be related to vitamin C metabolism, which has been shown to be altered in the CSF of patients with mild cognitive impairment and Alzheimer's disease (49). Creatine is routinely measured in brain magnetic resonance spectroscopy studies of aging (50), supporting its relevance to brain energy metabolism, although its alteration in CSF remains less consistently documented. Sarcosine, a glycine-related metabolite, has not been directly reported in the above brain aging studies to the best of our knowledge. In contrast, nepodin, chlorophene, resorcinol, isethionate, and Fa 18:2 + 1O were not reported in prior brain aging metabolomics datasets and may represent novel candidates. However, because some of these metabolites, particularly chlorophene, resorcinol, and isethionate, could have exogenous origins or reflect medication use, additional validation in independent cohorts with exposure data is warranted.

Although untargeted metabolomics is widely applied recently, metabolite profiles are affected by many confounding factors, which may lead to the instability of results. In this study, medication use, diet, and lifestyle factors are controlled when participants are included. However, the influence of pre-surgical fasting and anesthetic procedures on metabolite profiles is not considered in this study. Floegel et al. have investigated fasting-induced metabolic changes and confirmed significant alterations in lipid and amino acid metabolism (43). Mazzeo et al. have studied the metabolic effects of anaesthesia and found disruptions in energy metabolism pathways (44). Taken together, pre-surgical fasting and anesthesia induce dynamic, interrelated changes in metabolite profiles, reflecting shifts in energy metabolism, oxidative stress, and organ function. These alterations can be accounted for in perioperative care and metabolomics research.

At last, we acknowledge that this study still has several limitations. First, the sample size is not big enough due to the difficulties in recruiting enough participants and obtaining clinical CSF samples. We are still collecting more specimens. Future studies will be verified from various aspects including expanding the sample size, implementing k-fold CV and independent validation cohorts, and targeted detection. Therefore, our findings should be interpreted as exploratory and hypothesis-generating, warranting validation in more homogeneously matched cohorts. Second, although it is a challenging for us to collect enough CSF samples, potential impacts on result interpretation are hard to say. Third, there is a lack of independent validation of key findings, which will be performed by targeted metabolomics using a separate cohort in the following study. Despite these potential drawbacks, this study provides valuable insights into the metabolic changes in cerebrospinal fluid during brain aging through an untargeted metabolomics approach. The identification of 33 differentially accumulated metabolites and their associated pathways offers new perspectives for understanding normal brain aging mechanisms and age-related diseases.

## Conclusions

In conclusion, this untargeted metabolomics analysis identified 33 differentially accumulated CSF metabolites between younger and older surgical patients, suggesting age-associated metabolic differences in the cerebroventricular environment. The top enriched chemical classes were lipids and lipid-like molecules, organic acids and derivatives, and organoheterocyclic compounds, and the metabolites were potentially involved in pathways including ABC transporters, protein digestion and absorption, and glycine, serine, and threonine metabolism. However, owing to the small sample size, lack of independent validation, and multiple-testing limitations, these findings should be interpreted as exploratory and hypothesis-generating rather than as evidence of metabolic mechanisms of normal brain aging. Larger studies with independent validation are needed to confirm these candidate metabolites and clarify their potential relevance to age-related brain changes.

## Data Availability

The datasets presented in this study can be found in online repositories. The names of the repository/repositories and accession number(s) can be found in the article/Supplementary Material.

## Supplementary material

Supplementary Table S1

Baseline characteristics of patients.

Supplementary Table S2

Basic information of 33 candidate metabolites.
