# Supplementary material for: Cerebrospinal fluid metabolite alterations associated with human brain aging: an untargeted metabolomics study
Source: Front Med (Lausanne). 2026 Sep 28;13:1960863. doi: 10.3389/fmed.2026.1960863 (PMC13654730; doi:10.3389/fmed.2026.1960863)
Supplement: Supplementary file 1 [file Table1.docx]

*Pan et al. 16 September 2026*

**Supplementary Tables**

**Supplementary Table 1. Baseline characteristics of patients**

| **Variable** | **The aging group (n=10)** | **The control group (n=10)** | **SMD** | **p-value** |
| --- | --- | --- | --- | --- |
| Sample size, n | 10 | 10 |  |  |
| Anesthesia technique: combined spinal–epidural (CSE), n (%) | 10 (100.0%) | 10 (100.0%) | 0.00 | — |
| Perioperative antibiotic prophylaxis required, n (%) | 10 (100.0%) | 10 (100.0%) | 0.00 | — |
| Age (years), mean ± SD | 73.85 ± 2.73 | 30.20 ± 3.12 | 12.28 | 8.88e-16 |
| BMI (kg/m²), mean ± SD | 22.33 ± 0.90 | 22.32 ± 0.96 | 0.01 | 0.981 |
| Male, n (%) | 5 (50.0%) | 6 (60.0%) | -0.20 | 1.000 |
| Perioperative NSAID use, n (%) | 5 (50.0%) | 5 (50.0%) | 0.00 | 1.000 |
| Surgery category 1 (arthroplasty/prosthesis), n (%) | 10 (100.0%) | 3 (30.0%) |  |  |
| Surgery category 2 (fracture fixation/arthrodesis + implant), n (%) | 0 (0.0%) | 4 (40.0%) |  |  |
| Surgery category 3 (arthroscopy + implant/reconstruction), n (%) | 0 (0.0%) | 3 (30.0%) |  | 4.59e-03 |

Note: |SMD| < 0.10 indicates balance. P-values: Welch's t-test for continuous variables, Fisher's exact test for binary variables, and Pearson's chi-square test for the 3-category surgery variable. Fasting standardized to 8 h (solids) and 2 h (clear fluids). Antibiotic prophylaxis followed orthopedic implant guidelines (cefazolin first-line; vancomycin or clindamycin for severe β-lactam allergy or MRSA colonization; total duration ≤24 h).

**Supplementary Table 2. Basic information of 33 candidate metabolites**

| **Name** | **VIP** | **Fold change** | **95%CI** | **p-value** | **Q-value** |
| --- | --- | --- | --- | --- | --- |
| Nepodin | 1.81096157 | 0.259736846 | [0.1536, 0.5500] | 0.008599516 | 0.1403126435036496 |
| Fa 18:2+1o | 1.176364553 | 0.335619953 | [0.2497, 0.8529] | 0.044781158 | 0.25208460190668347 |
| L-threonate | 10.32939938 | 0.356675718 | [0.2060, 0.5820] | 0.002447861 | 0.10103821676712328 |
| Dimethylglycine | 1.081159984 | 0.369908673 | [0.2444, 0.6285] | 0.006568891 | 0.13209229362162161 |
| Glycolate | 2.078743594 | 0.42670133 | [0.2821, 0.6741] | 0.007234357 | 0.1362177852631579 |
| Homocarnosine | 1.52197556 | 0.447005079 | [0.2907, 0.6966] | 0.003060338 | 0.10812394510948904 |
| D-psicose | 10.17597818 | 0.482973887 | [0.2830, 0.7186] | 0.005494163 | 0.12639484729927006 |
| 3-methyl-2-oxopentanoate | 3.120605517 | 0.485141714 | [0.3480, 0.7512] | 0.006006431 | 0.12692089123404254 |
| L-aspartic acid | 4.497959368 | 0.506136664 | [0.1697, 0.7518] | 0.0022056852 | 0.19267929196875 |
| Chlorophene | 3.279124731 | 3.122839796 | [1.8263, 6.8084] | 0.004488728 | 0.11849039162790698 |
| Resorcinol | 3.382365659 | 1.845376353 | [1.0033, 14.4312] | 0.01789684 | 0.178142786551495 |
| Isethionate | 10.11241045 | 1.738579388 | [1.2647, 2.6486] | 4.28393e-4 | 0.08100124416 |
| Phytanic acid | 2.487534262 | 1.604077313 | [1.2603, 2.2403] | 5.27753e-4 | 0.0853712897142857 |
| Orcinol | 3.364217296 | 1.590120445 | [1.1494, 2.5458] | 0.018082134 | 0.17858107561061948 |
| Citramalic acid | 1.898369595 | 1.574302494 | [1.0567, 2.7944] | 0.030701212 | 0.21747772548770816 |
| Octadecanoic acid | 16.58360349 | 1.452162273 | [1.2128, 1.8181] | 3.55402e-4 | 0.07554831085714286 |
| Palmitic acid | 14.3870776 | 1.375806215 | [1.1519, 1.6918] | 0.002090263 | 0.09786445236363636 |
| Rhamnetin | 1.901004799 | 1.133178732 | [0.9943, 1.3208] | 0.033873956 | 0.22535520057228017 |
| Didodecyl 3,3’-thiodipropionates | 4.934648447 | 0.267061913 | [0.1237, 0.6256] | 0.003632654 | 0.1034566239491917 |
| Syringic acid | 3.02567298 | 0.267384256 | [0.1393, 0.3773] | 5.6982e-7 | 0.0035015438999999996 |
| Alpha-d-mannose pentaacetate | 1.039559387 | 0.289287927 | [0.1105, 0.5406] | 0.013000057 | 0.14572276526891523 |
| Zerumbone | 3.009439383 | 0.320722129 | [0.1462, 0.6741] | 0.043689459 | 0.2324430524285714 |
| Leucine | 4.88346782 | 0.354954684 | [0.2313, 0.6696] | 0.018257186 | 0.1649529971010249 |
| L-propionylcarnitine | 1.29254906 | 0.358888002 | [0.0667, 1.0706] | 0.0311785 | 0.20106369716159495 |
| Allose | 1.62645907 | 0.410476787 | [0.2116, 0.7615] | 0.021171265 | 0.17369482433244327 |
| Tebutam | 8.998267453 | 0.412590188 | [0.1840, 0.9754] | 0.013651793 | 0.14932028535587188 |
| Diethylcarbamazine | 7.134566741 | 0.416772052 | [0.2411, 0.6736] | 9.31402e-4 | 0.08517130572864322 |
| Creatine | 11.07033261 | 7.309859121 | [2.2526, 202.2593] | 0.014692434 | 0.15254048218776195 |
| Sarcosine | 2.468587633 | 4.175785134 | [1.9085, 31.5417] | 0.004904917 | 0.10813358694642858 |
| N-stearoylsphinganine | 4.644089962 | 1.699601237 | [1.3101, 2.4518] | 1.9556e-4 | 0.06825393307692308 |
| N-acetyl-l-aspartic acid | 2.267392985 | 1.672227781 | [1.1398, 2.8569] | 0.0067689 | 0.1208394821602289 |
| Alisol a | 2.264524162 | 1.324256596 | [0.9767, 1.7061] | 0.044441616 | 0.23424439074185247 |
| 3-phosphonoalanine | 1.751327358 | 1.226851814 | [1.0082, 1.4988] | 0.028441665 | 0.19294878377202643 |
| 3,5-dibromo-l-tyrosine | 1.339089447 | 1.099510198 | [1.0457, 1.1603] | 5.72734e-4 | 0.08184768441860464 |
